# Childhood Abuse and Delinquency: A Descriptive Study of Institutionalized Female Youth in Singapore

**DOI:** 10.1080/13218710802552971

**Published:** 2009-04-23

**Authors:** Chi Meng Chu, Stuart D. M. Thomas, Vivienne P. Y. Ng

**Affiliations:** ^a^ Centre for Forensic Behavioural Science, Monash University, Melbourne, Australia; ^b^ Psychological Services Unit, Ministry of Community Development, Youth and Sports, Singapore

**Keywords:** adolescents, child abuse, delinquency, female, institutionalization, multiple abuse, psychological adjustment

## Abstract

Childhood abuse experiences appear prevalent in institutionalized children and youth, but research to date has been limited. Moreover, there is no published study that has examined the issue of childhood abuse and delinquency within institutionalized children and youth in Singapore. This study was cross-sectional in design with follow-up criminal record checks. Data were collected from multiple data sources including official records, structured clinical interviews, and self-report questionnaires on 79 adolescent female residential youth. Two thirds reported having experienced childhood abuse and a higher proportion reported having previously engaged in self-harming behaviours. Participants with multiple types of abuse reported being abused at a younger age, were more likely to be subjected to repeated physical abuse, to have overdosed on medication, and to have reported more health and sexual concerns and trauma symptomatology than those who reported either experiencing one or no previous types of abuse. Fourteen (18%) of the sample had been convicted of further criminal offences over a 6½-year follow-up. The adolescents with histories of multiple types of abuse in childhood presented with more health concerns and traumatic symptomatology, self-reported delinquency, as well as past self-harming behaviour during their institutional stay. Identification of these additionally vulnerable adolescents has important clinical implications for identification, assessment and intervention planning.

Childhood abuse has been associated with long-term detrimental effects, whereby the more severe the abuse, the greater the probability of psychiatric disorder (Fergusson and Lynskey, 1997). The associated psychopathology in adulthood includes behavioural (e.g., Egeland, Yates, Appleyard, and van Dulmen, 2002), emotional (e.g., Danielson, De Arellano, Kilpatrick, Saunders, and Resnick, 2005), psychological (e.g., Noll, Horowitz, Bonanno, Trickett, and Putnam, 2003), and relationship problems (e.g., DiLillo and Long, 1999). Research also consistently reports that childhood abuse is associated with trauma symptomatology in adulthood (Boney-McCoy and Finkelhor, 1996; Messman-Moore and Brown, 2004; Schaaf and McCanne, 1998, Silverman, Reinherz, and Giacona, 1996).

In addition, victims of abuse often appear to experience a pattern of continual and multiple victimizations (e.g., Finkelhor, Ormrod, and Turner, 2007a). Published rates of co-occurring physical and sexual abuse vary widely between different settings: from 17% in a community sample of children (Hobbs and Wynne, 1990) to upwards of 71% in samples of inpatient adolescent girls with mental health needs (Westen, Ludolph, Misle, Ruffins, and Block, 1990). Studies have found that multiple types of victimization are associated with more severe abuse and trauma symptomatology (Clemmons, Walsh, DiLillo, and Messman-Moore, 2007; Finkelhor et al., 2007a), more delinquent behaviours (Arata, Langhinrichsen-Rohling, Bowers, and O'Farrill-Swails, 2005), and poorer psychosocial adjustment and outcomes (Clemmons, DiLillo, Martinez, DeGue, and Jeffcott, 2003).

## Childhood Abuse and Delinquency in Institutionalized Children and Youth

Studies report high rates of abuse among institutionalized children and youth, with between 57% and 80% reporting having experienced physical abuse; between 47% and 54%, child sexual abuse; and between 61% and 78% having been neglected (Brady and Caraway, 2002; Holland and Gorey, 2004; Hussey and Guo, 2002). More generally, Weine, Becker, Levy, Edell, and McGlashan (1997) observed that 81% of their sample of 75 institutionalized youth had experienced at least one form of traumatic event during their lifetime. Other studies have shown that mood disorders, anxiety, anger, sexual concerns, behavioural concerns, substance use, and post-traumatic stress symptoms are common among institutionalized children and youth who have been abused (Brady and Caraway, 2002; Hibbard, Spence, Tzeng, Zollinger, and Orr, 1992; Weine et al., 1997). Furthermore, there is some suggestion of an association between childhood abuse and subsequent delinquent behaviour among institutionalized children and youth. For example, in a longitudinal study of institutionalized adolescent boys, Zeiller (1982) reported that physically and psychologically abused youth were three times more aggressive than their not-abused peers, and twice as likely to subsequently engage in some form of delinquent behaviour.

## Singaporean Context

Singapore is a small independent island-state in South East Asia. Its population of 4.68 million consists of 74.8% Chinese, 13.5% Malay, 9% Indian, and 1.7% other ethnicities (Singapore Department of Statistics, 2007). Previous studies of physical and sexual abuse survivors in Singapore have found that the majority are children and youth of school age (Chan, 1987; Tong, Elliot, and Tan, 1996). Although the majority of the physical abuse perpetrators were the natural parents of the child victims, non-caregivers appeared to account for most of the sexual abuse perpetrators in Singapore (Tong et al., 1996). In addition, comparisons between US and Singaporean female samples suggest the presence of cross-cultural differences in abuse experiences. Specifically, Back et al. (2003) found that female college students in Singapore with a history of child sexual abuse reported more severe psychological symptomatology relative to their non-abused peers and US counterparts.

## Present Study

To date there has been only limited research on childhood abuse and delinquency in institutionalized children and youth, and currently none within the Singaporean context. Due to the dearth of extant evidence in this area, this study adopted a descriptive, exploratory approach. Its aims were: (a) to explore the characteristics of the female youth who were institutionalized in Singapore, (b) to examine their childhood abuse experiences, (c) to examine current functioning within the institution, and (d) to examine the rates of reoffending among the sample.

## Method

### Source Sample

The eligible source sample consisted of all female residents admitted to an adolescent female residential facility in Singapore, for either delinquency or protection reasons, between May and July 2001. Potential participants were excluded only if they were engaged in educational or community re-integration programmes primarily that did not permit sufficient time for meaningful participation in the research.

### Ethics

Ethical approval was obtained through the Psychological Services Unit and the Director of Rehabilitation, Protection, and Residential Services Division of the Ministry of Community Development, Youth and Sports before the commencement of the study in 2001. Informed consent was sought from all participants and the limits to confidentiality were explained (e.g., threat of harm to self and others).

### Procedure

Information was collected from multiple data sources including official records, structured clinical interviews, and self-report questionnaires. A pro forma was designed specifically for use in the study to code the information obtained from the clinical interview, official records, and self-report questionnaires. The major coding categories were as follows: (a) sociodemographic characteristics: age, education level, ethnicity, and family structure, (b) residential stay information: date of admission, age at admission, length of stay, and reason for committal in the residential facility (for delinquency vs. protective reasons), (c) abuse experiences information: type of abuse (physical, sexual, or both), age when first abused, duration of abuse, number of perpetrators (single or multiple), and relationship to perpetrators (familial vs. non-familial), (d) self-harm/suicidal behaviours: self-harm behaviours included inflicting bodily pain and medication overdose, whereas suicidal behaviours included suicidal ideations and suicidal attempts, and (e) psychometric data: subscale and total scores from standardized self-report questionnaires (see below).

This study was cross-sectional in design with criminal record checks completed at the end of 2007. The third author (VN), together with a senior psychologist and two research assistants, administered the structured clinical interview and self-report questionnaires to participants. The research assistants were given 1 hr of training in relation to the administration of the structured clinical interview and questionnaires prior to the commencement of the study. Discrepancies between data sources were resolved by referring to the interview findings.

Sociodemographic data were obtained from official records and via interviews; any discrepancies were clarified with participants. Three classifications were used for parental marital status: (a) natural intact (i.e., parents were married); (b) divorced/separated; (c) deceased (i.e., either one or both parents were deceased).

Information relating to the residents' stay in the facility, as well as their self-harm and suicidal behaviours were coded from official records and clarified with the participants during the interview. The participants' abuse experiences were coded from the structured interviews conducted with all participants. Specifically, the type of abuse was classified as “physical abuse” if it involved physical battery or assault (e.g., hitting, kicking, punching, stabbing, choking, burning, throwing against wall, and pulling of hair) of the participants without sexual victimization, whereas “sexual abuse” was defined as inappropriate and/or unwanted sexual experiences that involved molestation, as well as anal, oral and vaginal penetration. Abuse was classified as “poly abuse” if it involved physical and sexual abuse. The repetitiveness of the abuse was coded as “repeated incidents” if there were more than three abuse episodes and “isolated incidents” if otherwise. The relationship with abuse perpetrator was classified as either “familial” or “non-familial” according to whether the perpetrator was a family member or relative; friends, classmates, and strangers were classified as non-familial perpetrators.

### Measures

The participants completed the following psychometric measures.

1. General Health Questionnaire-28 (GHQ-28) (Goldberg and Williams, 1988). The GHQ-28 is a well-established 28-item self-report measure used to detect psychiatric disorder in the general population and within community or non-psychiatric clinical settings such as primary care or general medical outpatients. GHQ-28 has four subscales that assess somatic symptoms, anxiety/insomnia, social dysfunction, and severe depression. Specifically, the raw total and subscale scores were considered here.
2. Reynolds' Adolescent Depression Scale–Second Edition (RADS-2) (Reynolds, 2002). The RADS-2 is a brief and established self-report measure that evaluates the current level of depressive symptomatology according to four subscales: dysphoric mood, anhedonia/negative affect, negative self-evaluation, and somatic complaints. Raw scores were considered in all analyses.
3. Trauma Symptom Checklist for Children (TSCC) (Briere, 1996). The TSCC is a widely used self-report measure of post-traumatic distress and related symptoms in male and female children aged 8–16 years. It consists of six subscales that assess the domains of anxiety, depression, anger, post-traumatic stress symptoms, dissociation and sexual concerns. Raw scores were used in all analyses.
4. Youth Self-Report (YSR) (Achenbach and Rescorla, 2001). The YSR is a reliable and validated self-report questionnaire designed to obtain 11–18-year-olds' reports of their own competencies and problems (e.g., withdrawn and anxious/depressed behaviours, social, thought and attention problems, as well as aggressive and delinquent behaviours). A modified YSR, with only the aggressive and delinquent behaviours subscales, was administered to the participants in this study.

### Statistical Analyses

The sample was characterized using simple descriptive statistics, with categorical data being reported as numbers and percentages, and continuous data presented in relation to the mean and standard deviation. Histograms of the continuous data were plotted to check for skewed distributions. Univariate analyses sought to compare the characteristics of the physically abused, sexually abused and poly-abused participants. Chi-square tests of association were computed for categorical data, while analyses of variance (ANOVA) was utilized for continuous data. Analyses were carried out using SPSS version 15.0.

## Results

### Characteristics of Residents

Ninety-nine female youth resided in the institution during the 3-month period. Twenty residents did not participate in the study due to conflicting rehabilitation schedules. All remaining residents consented to participation in the study; the source sample therefore consisted of 79 female youth, admitted mainly for delinquency reasons (68/79, 86%).

The mean age of the residents on admission and at the time of interview was 14.92 years (*SD* = 1.62, range = 11–19 years), and 15.99 years (*SD* = 1.62, range = 13–20 years), respectively, and was normally distributed. Their length of stay within the institution was positively skewed, with the shortest stay being that of a girl on her first day of admission, and the longest length of stay being 1,015 days at the time of interview (*M* = 390 days, *SD* = 223, *Mdn* = 371 days). The sample largely consisted of Chinese (51%) and Malay (38%) subjects, and the majority (70%) had received at least secondary-level education. In addition, the majority came from natural intact (53%) or divorced/separated families (39%).

Table 1 summarizes the victimization experiences of the residents. More than two thirds (52/79) had past victimization experiences, and almost one quarter (19/79) had experienced poly-abuse (i.e., both sexual and physical abuse). Of the residents who experienced one type of abuse, 64% (21/33) experienced only physical abuse and 36% (12/33) reported being sexually abused. The poly-abused girls were more likely than the singly abused to be first abused at an earlier age, *t*(49) = 2.14, *p* = .037, two-tailed.

**Table:** Table 1. Residents' victimization experiences.

	SAMPLE *N*^†^ (%)
Victimization experience	
Non-abused	27/79 (34)
Singly abused (PA or SA)	33/79 (42)
PA	21/33 (64)
SA	12/33 (36)
Poly-abused (PA and SA)	19/79 (24)
Frequency of abuse	
PA only: Isolated incidents	17/21 (81)
PA only: Repeated incidents	4/21 (19)
SA only: Isolated incidents	9/11 (82)
SA only: Repeated incidents	2/11 (18)
Poly-abuse	PA/SA
Isolated incidents	8/18 (44)/13/18 (72)
Repeated incidents	10/18 (56)/5/18 (28)
Perpetrators of abuse	
Familial	28/52 (54)
Non-familial	24/52 (46)
No. perpetrators	
Single	22/39 (56)
Multiple	17/39 (44)
	Mean (*SD*)/Range
Age when first abused (years)	
All abused (*n* = 51)	11.29 (3.58)/1–16
Singly abused (*n* = 32)	12.09 (3.53)/3–14
Poly-abused (*n* = 19)	9.95 (3.33)/1–16
Duration of abuse (years)	
PA (*n* = 39)	2.90 (3.74)/0–14
SA (*n* = 26)	1.88 (2.07)/0–8
Duration from last recalled abuse (years)	
All abused (*n* = 51)	1.88 (1.58)/0–7
Singly abused (*n* = 32)	1.68 (1.60)/0–5
Poly-abused (*n* = 19)	2.00 (1.57)/0–7
*Notes*. PA = physical abuse; SA = sexual abuse.	
^†^Differences in the denominators are due to missing data.	

More than two thirds (55/78) of the sample had a history of self-harming behaviour, and almost one quarter (18/79) had past suicidal attempts. Approximately two thirds (51/79) had inflicted bodily pain when self-harming, which was the most common type of self-harm behaviour, while just under one third (24/78) reported having previously overdosed on medication (Table 2).

**Table:** Table 2. Residents' self-harm behaviours.

	SAMPLE *N*^†^ (%)
Self-harm behaviour	
Has previous self-harm behaviour(s)	55/78 (71)
No self-harm behaviour	23/78 (29)
Types of self-harm behaviour	
Inflict bodily pain	51/79 (65)
No inflicting of bodily pain	28/79 (35)
Overdose on medication	24/78 (31)
No overdose on medication	54/78 (69)
Suicidal ideation	
Has previous suicidal ideation	40/79 (51)
No suicidal ideation	39/79 (49)
Active suicidal ideation (at time of interview)	8/79 (10)
No active suicidal ideation	71/79 (90)
Suicide attempt	
Has previous suicidal attempt(s)	18/79 (23)
No suicide attempt	61/79 (77)
^†^Differences in the denominators are due to missing data.	

### Recidivism

During a 6½-year-follow-up (until end 2007), 18% (14/79) were reconvicted of further criminal offences. There were no statistical differences between the characteristics of the recidivists and non-recidivists. Type of abuse experienced did not predict reconviction, χ^2^(1,*N*=79) = 0.49, *p* = .81, with only three of the recidivists being classified as poly-abused.

### General Functioning of the Poly-Abused Versus Others

When the poly-abused were compared with the singly abused and non-abused with respect to the ratings on the standardized measures, a number of differences arose. The poly-abused group recorded significantly higher scores on GHQ-28, *F*(2,76) = 5.01, *p* = .009, and YSR Delinquency Scale, *F*(2,72) = 4.91, *p* = .01. They also scored significantly higher on TSCC Anger, Post-traumatic Symptoms, and Sexual Concern scales than the two other groups, *F*(2,72) = 8.37, *p* = .001, *F*(2,72) = 4.52, *p* = .014, and *F*(2,72) = 3.71, *p* = .029, respectively. Although there were no differences with respect to depressive symptoms on TSCC Depression, *F*(2,72) = 1.99, *p* = .145, those who were poly-abused scored significantly higher total scores on the RADS-2 depression scale than the other groups, *F*(2,76) = 3.75, *p* = .028. This group was also more likely to have overdosed on medication previously, χ^2^(1,*N*=79) = 5.64, *p* = .018, and more likely to have been abused at a younger age, *F*(1,49) = 4.60, *p* = .037; as well as being abused repeatedly, χ^2^(1,*N*=79) = 5.61, *p* = .018.

## Discussion

Childhood abuse has been associated with a myriad of debilitating long-lasting effects. For institutionalized youth, such abuse may represent an ongoing social, clinical and policy issue for health and justice services in Singapore as well as international contexts. This study describes the characteristics of 79 female youth who were residing within a juvenile institution during a 3-month period in 2001.

Before we discuss the findings, there are several limitations that may impact on the generalizability of the study's findings. First, the current symptomatology present in the participants who had a history of abuse may not necessarily be a result of their childhood trauma. For example, the symptomatology could be at least in part a result of their other experiences, whether currently or in the past. Clearly, it will be useful to distinguish the contribution of recent victimization experiences from that of the past. Second, the present study was cross-sectional in nature; whereby the single point of assessment may not accurately reflect the participants' functioning during their institutional stay. Third, the information recorded and considered here was based on self-report and did not have multi-informant corroboration, which would provide richer (and more accurate) information regarding the participants' histories (substance abuse, victimization and delinquency), as well as their trauma symptomatology. Finally, the study did not collect data on (a) the circumstances surrounding the abuse, (b) other types of abuse (e.g., psychological, emotional, and neglect), (c) family environment, or (d) the victimization experiences in the residential institution. Notwithstanding the aforementioned limitations, this study describes the characteristics of what equates to a sizeable number of institutionalized female youth, within a small nation such as Singapore. Collectively, these findings potentially have important implications for clinical practice and further research.

Two thirds of the participants reported having experienced at least one type of childhood abuse, and one quarter had experienced multiple types of childhood abuse. When compared to the institutionalized youth with abuse experiences in Western countries (Brady and Caraway, 2002; Holland and Gorey, 2004; Hussey and Guo, 2002; Weine et al., 1997; Westen et al., 1990), sexual and physical abuse appeared to be generally less prevalent within this sample. With regard to exposure to traumatic events, there is a substantial discrepancy between the prevalence rates across different studies. In this study one quarter of the sample reported both physical and sexual abuse experiences; as compared to 88% of a relatively younger (*M* = 9.8 years, *SD* = 1.74) US sample of institutionalized children and youth who had experienced at least two types of traumatic events (Brady and Caraway, 2002). One reason for such a large discrepancy may be the different operationalizations of the victimization experiences between studies. The standard use of multiple data sources to clarify any discrepancies between self-reports and official records, as well as further consideration of other forms of abuse (e.g., psychological, emotional, neglect, and witness of domestic violence) may provide a more comprehensive and therefore more accurate picture to better inform subsequent international and cross-cultural comparisons.

More than two thirds of the participants had a history of self-harm behaviours and almost one quarter reported previous suicidal attempts. Although more than half reported previous suicidal ideation, one in 10 were actively thinking of attempting suicide at the time of interviews, thereby suggesting an additionally vulnerable group presenting significant care and treatment concerns for residential service providers. It was noted that the residents with sexual abuse and poly-abuse histories were more likely to have a history of suicidal attempts and overdose on medication, respectively. The data were not forthcoming about the precise attributions for these ideations and behaviours, but these research findings clearly highlight the need for continual, proactive assessment and provision of care and support to this particularly vulnerable group of institutionalized youth during periods of institutionalization and upon release, and facilitation of community reintegration strategies to improve health-related outcomes.

It was observed that the poly-abused youth were abused at a younger age than the singly abused. Although not necessarily a result of their victimization experiences, the general functioning of the poly-abused group was lower than singly abused and non-abused. The poly-abused exhibited more trauma symptomatology, negative emotions, health concerns and sexual concerns than other groups, which is consistent with other studies on multi-victimization survivors (e.g., Clemmons et al., 2007; Finkelhor et al., 2007a). The poly-abused subjects were also more likely to overdose on medication when self-harming.

It is, however, interesting to note that the singly abused subjects did not differ significantly from the non-abused, but were significantly different from the poly-abused subjects in terms of trauma symptomatology, delinquency, and self-harm behaviours. Specifically, what had accounted for the lack of differences, in terms of trauma symptomatology and outcomes, between singly abused and the non-abused subjects remains unclear from data that arose from what was a very structured, mechanistic interview process. Unfortunately the present findings, albeit seemingly consistent with the Finkelhor et al. (2007a) assertion of the dramatic elevations in symptomatology due to accumulation of victimization experiences, could not explain the lack of incremental symptomatology from the non-abused to singly abused groups. This therefore remains an area for further in-depth qualitative exploration and consideration.

Follow-up recidivism checks after 6½ years (until end 2007) showed that almost one fifth of the participants had been charged with and convicted of further criminal offences. Of note, although those who reported having been poly-abused self-reported more delinquent behaviours than other groups, official follow-up of criminal convictions did not suggest any statistically significant differences between groups. Several reasons could account for this finding: (a) a number may have reoffended but not been caught, (b) some may have reoffended but had not accrued a further conviction by the end of the follow-up period, or even perhaps that (c) treatment during the institutional stay could have positively impacted upon outcomes in the short to medium term. While it is fair to say that the official rates of subsequent criminal behaviour are undoubtedly a dramatic underestimate of the frequency of such behaviour in this, and other similar, populations; the veracity and comprehensiveness of self-report indices of these criminal behaviours do not lend themselves to be an entirely valid alternative. A further, more detailed, triangulation of self-reported and collateral rates of offending coupled with official record documentation may help further explain this continuing debate.

The current study provides a much-needed picture of the characteristics and general functioning of institutionalized female youth in Singapore. One arising clinical issue of concern is the identification of a group of residents that have multi-victimization histories. If identified early enough, appropriate treatment and monitoring could help them address and/or alleviate emotional and behavioural difficulties during their institutional stay. This is particularly important given the recent findings that multi-victimized survivors are at increased risk of persistent multi-victimization, with those children and youth with higher anger/aggression being particularly vulnerable (Finkelhor et al., 2007b). It would also be prudent to continue to monitor and provide support to these survivors to facilitate channelling into appropriate treatment and other supportive programmes to help prevent further progression on the victimization ladder. Ultimately, such interventions would have the potential to translate into better institutional adjustment, as well as more positive longer-term health and justice-related outcomes.
